# Laboratory Findings Associated With Severe Illness and Mortality Among Hospitalized Individuals With Coronavirus Disease 2019 in Eastern Massachusetts

**DOI:** 10.1001/jamanetworkopen.2020.23934

**Published:** 2020-10-30

**Authors:** Victor M. Castro, Thomas H. McCoy, Roy H. Perlis

**Affiliations:** 1 Center for Quantitative Health, Division of Clinical Research, Massachusetts General Hospital, Boston, Massachusetts; 2 Research Information Science and Computing, Mass General Brigham, Somerville, Massachusetts

## Abstract

**Importance:**

The coronavirus disease 2019 (COVID-19) pandemic has placed unprecedented stress on health systems across the world, and reliable estimates of risk for adverse hospital outcomes are needed.

**Objective:**

To quantify admission laboratory and comorbidity features associated with critical illness and mortality risk across 6 Eastern Massachusetts hospitals.

**Design, Setting, and Participants:**

Retrospective cohort study of all individuals admitted to the hospital who tested positive for severe acute respiratory syndrome coronavirus 2 (SARS-CoV-2) by polymerase chain reaction across these 6 hospitals through June 5, 2020, using hospital course, prior diagnoses, and laboratory values in emergency department and inpatient settings from 2 academic medical centers and 4 community hospitals. The data were extracted on June 11, 2020, and the analysis was conducted from June to July 2020.

**Exposures:**

SARS-CoV-2.

**Main Outcomes and Measures:**

Severe illness defined by admission to intensive care unit, mechanical ventilation, or death.

**Results:**

Of 2511 hospitalized individuals who tested positive for SARS-CoV-2 (of whom 50.9% were male, 53.9% White, and 27.0% Hispanic, with a mean [SD ]age of 62.6 [19.0] years), 215 (8.6%) were admitted to the intensive care unit, 164 (6.5%) required mechanical ventilation, and 292 (11.6%) died. L1-regression models developed in 3 of these hospitals yielded an area under the receiver operating characteristic curve of 0.807 for severe illness and 0.847 for mortality in the 3 held-out hospitals. In total, 212 of 292 deaths (72.6%) occurred in the highest-risk mortality quintile.

**Conclusions and Relevance:**

In this cohort, specific admission laboratory studies in concert with sociodemographic features and prior diagnosis facilitated risk stratification among individuals hospitalized for COVID-19.

## Key Points

**Question:**

How well can sociodemographic features, laboratory values, and comorbidities of individuals hospitalized with coronavirus disease 2019 (COVID-19) in Eastern Massachusetts through June 5, 2020, predict a severe illness course?

**Findings:**

In this cohort study of 2511 hospitalized individuals positive for severe acute respiratory syndrome coronavirus 2 (SARS-CoV-2) by polymerase chain reaction who were admitted to 1 of 6 hospitals, 215 (8.6%) were admitted to the instensive care unit, 164 (6.5%) required mechanical ventilation, and 292 (11.6%) died. In a risk prediction model, 212 deaths (78%) occurred in the top mortality-risk quintile.

**Meaning:**

Simple prediction models may assist in risk stratification among hospitalized patients with COVID-19.

## Teaser

This cohort study assesses admission laboratory and comorbidity features associated with critical illness and mortality risk among patients hospitalized with coronavirus disease 2019 (COVID-19) across 6 Eastern Massachusetts hospitals.

## Introduction

With the rapid spread of coronavirus disease 2019 (COVID-19), efforts to predict clinical outcomes and stratify risk have taken on greater urgency as a means of allocating resources and targeting interventions. A recent report of 1099 admitted individuals from China found that 5.0% required intensive care unit (ICU) transfer, and 2.3% required mechanical ventilation.^1^ In Lombardy, Italy, approximately 16% of test-positive individuals required ICU admission.^2^ In the United States, the characteristics of admitted patients may differ somewhat. A recent case series from the Seattle area in Washington described 24 ICU-admitted patients, of whom 75% required mechanical ventilation.^3^ In one of the largest US studies, to date, among a series of 2634 hospitalized patients in New York, New York, who died or were discharged from the hospital, 12.2% had required mechanical ventilation.^4^

Given the constrained resources for treatment of COVID-19, particularly with regard to mechanical ventilation, simple approaches to stratifying morbidity and mortality risk at time of hospitalization are needed. In cohorts ranging from 100 to 200 patients, multiple laboratory studies have been associated with mortality risk, including elevated ferritin, troponin, and C-reactive peptide levels,^5^ elevated D-dimer level,^6^ and low eosinophil count.^7^ Most recently, a meta-analysis including a total of 3377 patients identified multiple blood cell indices as most strongly predictive of mortality.^8^

Electronic health records may facilitate a rapid and efficient investigation of clinical cohorts and may form the basis of efforts by consortia to address COVID-19 at scale.^9^ Here, we examined records from 2 academic medical centers and 4 affiliated community hospitals in Eastern Massachusetts. We applied data from 3 of these hospitals to generate simple and transparent models to estimate the risk of a severe hospital course, characterized by the need for mechanical ventilation, ICU level of care, or risk for death, and we validated these results in 3 held-out hospitals, including another academic medical center and 2 community hospitals, as a starting point for generalizable efforts at clinical risk stratification.^10^ We hypothesized that clinical and laboratory data available at data collection could efficiently stratify risk for a more severe in-hospital course or death.

## Methods

### Patients

The full cohort included all individuals aged 18 of age or older hospitalized at any of the 2 academic medical centers and 4 community affiliate hospitals between March 1 and June 5, 2020, with a documented polymerase chain reaction–positive test result for severe acute respiratory syndrome coronavirus 2 (SARS-CoV-2) within 5 days of admission. We excluded patients with a severe outcome on the same day of admission (for whom a prediction based on laboratory studies would be uninformative) and patients transferred from outside hospitals. For all of these individuals, data on prior diagnosis and course during the admission were extracted from the Partners Research Patient Data Registry^11^ and the Enterprise Data Warehouse and used to generate an i2b2 datamart.^12^ Data were augmented with age, sex, race, and race/ethnicity from the same source. The enterprise laboratory feed was used to extract SARS-CoV-2 test order and results, as well as additional laboratory values (eTable 1 in the Supplement). Laboratory values available in at least 80% of individuals were included in subsequent analysis as continuous measures, after Winsorization at the 99th percentile but otherwise without transformation, along with laboratory-specific high and low flags. As an aggregate measure of comorbidity, the Charlson comorbidity index was calculated using coded *International Classification of Diseases, Ninth Revision* (*ICD-9*) and *International Statistical Classification of Diseases and Related Health Problems, Tenth Revision* (*ICD-10*) diagnostic codes obtained from the electronic health record as previously described.^13^

The study protocol was approved by the Partners HealthCare Human Research Committee. No participant contact was required in this study, which relied on secondary use of data produced by routine clinical care, allowing for the waiver of a requirement for informed consent as detailed by 45 CFR 46.116. The Strengthening the Reporting of Observational Studies in Epidemiology (STROBE) reporting guideline for cohort studies was applied.

### Study Design and Statistical Analysis

We included all newly hospitalized individuals with a SARS-CoV-2–positive polymerase chain reaction test result within 5 days of admission. Patients were followed up from time of admission to hospital discharge or death, with follow-up censored at time of hospital discharge for consistency with similar publications and because mortality data are not yet available from independent sources (ie, National Death Index or equivalent). The 2 primary outcomes of interest were (1) COVID-19–associated severe illness, including any of the following: admission to the ICU, mechanical ventilation, or mortality, and (2) mortality. The former composite outcome was selected to avoid the problem of competing risk (ie, individuals with severe illness who die before either admission to the ICU or mechanical ventilation). We selected the earliest laboratory values and vital signs associated with the admission, including those measured in the emergency department. Prior *ICD* diagnoses were grouped using a Healthcare Utilization Project Clinical Classification Software hierarchy.^14^ The log-transformed counts of each Clinical Classification Software diagnosis group were used as predictors. Beyond descriptive analysis, we report appropriate univariate comparisons (ie, the χ^2^ test for binary variables and the *t* test for continuous measures) followed by L1-penalized logistic regression, or the least absolute shrinkage and selection operator (lasso),^15^ to identify a parsimonious model with sociodemographic features, baseline vital signs, prior diagnosis, and laboratory values as candidate predictors. The hospitals were divided into a training cohort (composed of 1 academic center and 2 community hospital) and an evaluative cohort (composed of the other 1 academic medical center and 2 community hospitals). The lasso was applied to all participants with complete laboratory studies in the training cohort and to the performance of the model evaluated in the wholly separate evaluative cohort. Model fitting used all individuals in the training set, with median imputation of missing data (including laboratory values); for individuals in the testing set, we substituted missing values with median values from the training set. Model performance was characterized using standard metrics of discrimination and calibration, focusing on the 5 quintiles of risk determined in the training data set, without recalibration.

These logistic regression models offer advantages in interpretability but fail to consider censoring. Therefore, to better characterize model performance in the testing set, for comparison we also used survival analysis, right-censoring at the time of hospital discharge or the end of available data (June 5, 2020), presenting Kaplan-Meier curves comparing risk quintile groups. All analyses used R, version 4.0.0.^16^ The log-rank test statistic was used to compare quintile curves.

## Results

The 2511 individuals hospitalized through June 5, 2020, included 1348 (53.7%) at academic medical centers and 1163 (46.3%) at community hospitals; 1277 (50.9%) were male, 1354 (53.9%) were White, and 679 (27.0%) were Hispanic; the mean (SD) age was 62.6 (19.0) years (Table 1). In all, 215 individuals (8.6%) were admitted to the ICU, 164 (6.5%) required mechanical ventilation, and 292 (11.6%) died. Of the 2511 total hospitalizations, 634 occurred in the testing cohort, and 1877 occurred in the training cohort. Laboratory values are summarized in eTable 2 and illustrated in eFigure in the Supplement.

**Table 1.** Sociodemographic Characteristics of Training and Test Sets

Characteristic	Patients, No. (%)			*P* value
	Training (n = 1877)	Test (n = 634)	Total (n = 2511)	
Hospital type				
Academic medical centers	992 (52.9)	356 (56.2)	1348 (53.7)	.15
Community hospitals	885 (47.1)	278 (43.8)	1163 (46.3)	
Age at admission, mean (SD), y	62.1 (19.3)	64.4 (18.0)	62.6 (19.0)	.007
Age group, y				
<30	99 (5.3)	22 (3.5)	121 (4.8)	<.001
30-39	189 (10.1)	50 (7.9)	239 (9.5)	
40-49	226 (12.0)	49 (7.7)	275 (11.0)	
50-59	309 (16.5)	111 (17.5)	420 (16.7)	
60-69	316 (16.8)	153 (24.1)	469 (18.7)	
70-79	324 (17.3)	102 (16.1)	426 (17.0)	
≥80	414 (22.1)	147 (23.2)	561 (22.3)	
Male sex	983 (52.4)	294 (46.4)	1277 (50.9)	.009
Race				
Asian	70 (3.7)	25 (3.9)	95 (3.8)	<.001
Black	209 (11.1)	219 (34.5)	428 (17.0)	
Other	318 (16.9)	71 (11.2)	389 (15.5)	
Unknown	175 (9.3)	70 (11.0)	245 (9.8)	
White	1105 (58.9)	249 (39.3)	1354 (53.9)	
Hispanic ethnicity	563 (30.0)	116 (18.3)	679 (27.0)	<.001
Charlson comorbidity index, mean (SD)	2.559 (3.254)	2.836 (3.607)	2.629 (3.348)	.07
ICU admission	161 (8.6)	54 (8.5)	215 (8.6)	.96
Mechanical ventilation	129 (6.9)	35 (5.5)	164 (6.5)	.23
Death	209 (11.1)	83 (13.1)	292 (11.6)	.18
COVID-19–associated severe outcome (ICU, mechanical ventilation, or death)	338 (18.0)	116 (18.3)	454 (18.1)	.87
Discharged to SNF or rehab	253 (40.6)	771 (42.1)	1024 (41.7)	.51

Abbreviations: COVID-19, coronavirus disease 2019; ICU, intensive care unit; rehab, rehabilitation; SNF, skilled nursing facility.

We used L1-penalized regression to train a prediction model based on admission characteristics, prior diagnosis, and laboratory values in 1 academic medical center and 2 community hospitals (Table 2). For severe illness, notable features included lymphocytopenia (low absolute lymphocyte count model coefficient: 0.3049), eosinopenia (eosinophil count model coefficient: −0.3129) and neutrophilia (high absolute neutrophil count model coefficient: 0.2332) as well as markers of diminished renal function (blood urea nitrogen model coefficient: 0.0091; high creatinine level model coefficient: 0.2546; low glomerular filtration rate model coefficient: 0.0736) (Table 2). For mortality (Table 2), the features were generally similar, with the addition of the presence of nucleated red blood cells (high absolute nucleated red blood cell count model coefficient: 0.3478) and other abnormal red blood cell indices (low mean corpuscular hemoglobin concentration model coefficient: 0.0099); red blood cell distribution width model coefficient: 0.0451), procalcitonin level (model coefficient: 0.1823), and greater representation of prior diagnosed codes consistent with pulmonary disease (prior diagnosis of COPD model coefficient: 0.0454; prior diagnosis of lung cancer model coefficient: 0.0389; prior diagnosis of respiratory failure model coefficient: 0.0373) (eTable 3 and eTable 4 in the Supplement report coefficients for features included in penalize regression, without shrinkage).

**Table 2.** Model Coefficients

Feature	Coefficients for COVID-19	
	Severe illness model	Mortality model
Age at admission	0.0129	0.0357
Baseline Spo_2_	−0.0009	−0.0107
Blood urea nitrogen level (continuous)	0.0091	0.0193
C-reactive protein level (continuous)	0.0014	Not included^a^
Charlson Comorbidity Index	0.0196	0.0137
Creatinine level (high)	0.2546	0.3607
Estimated glomerular filtration rate (low)	0.0736	0.0013
Eosinophil count (continuous)	−0.3129	−0.2550
Glucose level (continuous)	0.0015	Not included^a^
Lactate dehydrogenase level (continuous)	0.0031	0.0014
Lymphocyte count (continuous)	−0.0044	−0.0021
Absolute lymphocyte count (low)	0.3049	0.1523
Mean corpuscular hemoglobin concentration (low)	Not included^a^	0.0099
Monocyte count (low)	0.2437	Not included^a^
Neutrophil count (continuous)	0.0007	Not included^a^
Absolute neutrophil count (high)	0.2332	0.3655
Absolute nucleated red blood cell count (high)	Not included^a^	0.3478
Platelet count (continuous)	−0.0007	Not included^a^
Platelet count (low)	0.1195	0.2360
Prior diagnosis of respiratory infections (CCS 8.1)	0.0804	Not included^a^
Prior diagnosis of COPD or bronchiectasis (CCS 8.2)	Not included^a^	0.0454
Prior diagnosis of dementia/delirium (CCS 5.4)	Not included^a^	0.0366
Prior diagnosis of external causes of injury (CCS 18)	Not included^a^	0.0238
Prior diagnosis of lung cancer (CCS 2.2)	Not included^a^	0.0389
Prior diagnosis of respiratory failure/insufficiency (CCS 8.6)	Not included^a^	0.0373
Procalcitonin level (continuous)	Not included^a^	0.1823
Red blood cell distribution width (continuous)	Not included^a^	0.0451
Troponin T level (continuous)	0.0045	0.0030
White blood cell count (high)	Not included^a^	0.0064

Abbreviations: CCS, Clinical Classification Software; COPD, chronic obstructive pulmonary disease; COVID-19, coronavirus disease 2019; Spo_2_, oxygen saturation as measured by pulse oximetry.

^a^ The specific feature was not included in the model for the outcome.

**Figure 1.**
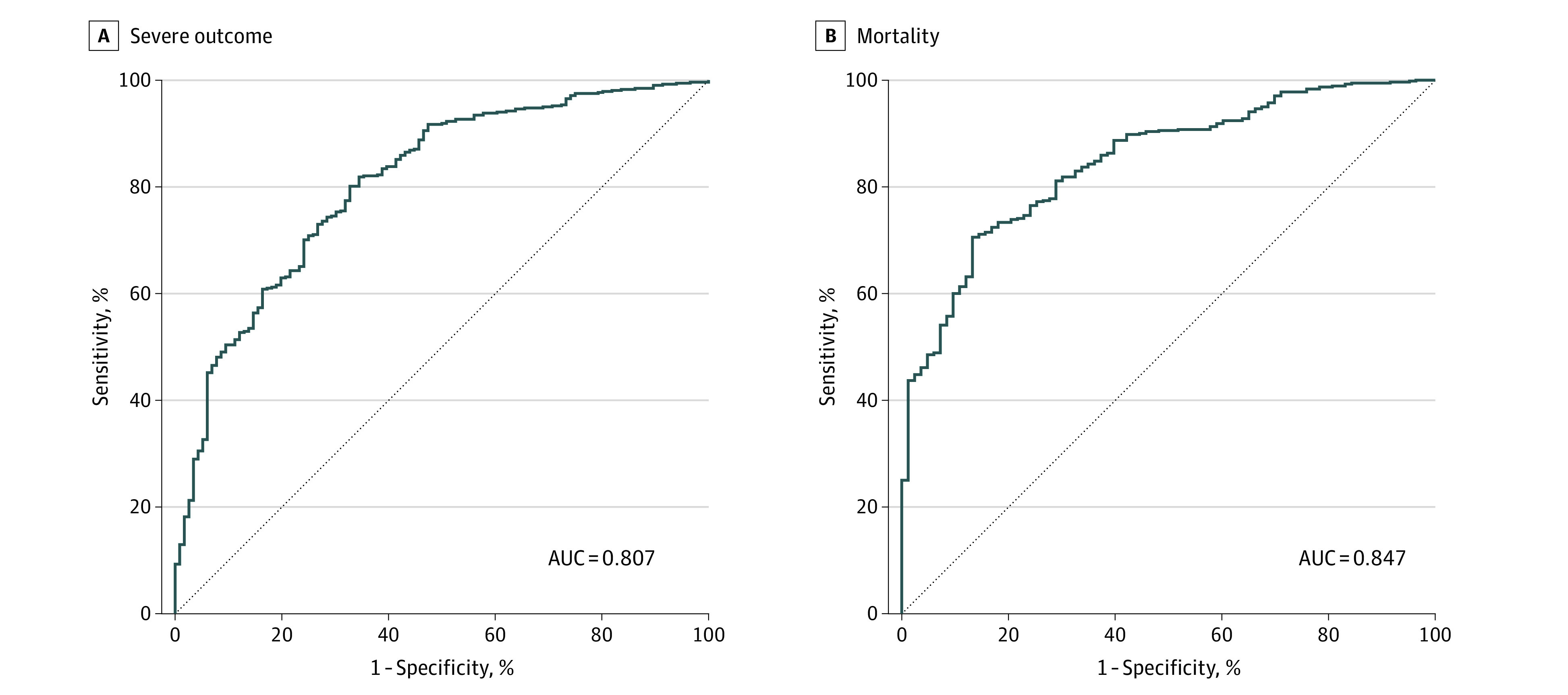
Model Performance in Test Set A, Coronavirus Disease 2019 (COVID-19)–associated severe outcome. B, COVID-19–associated mortality. AUC indicates area under the curve.

**Figure 2.**
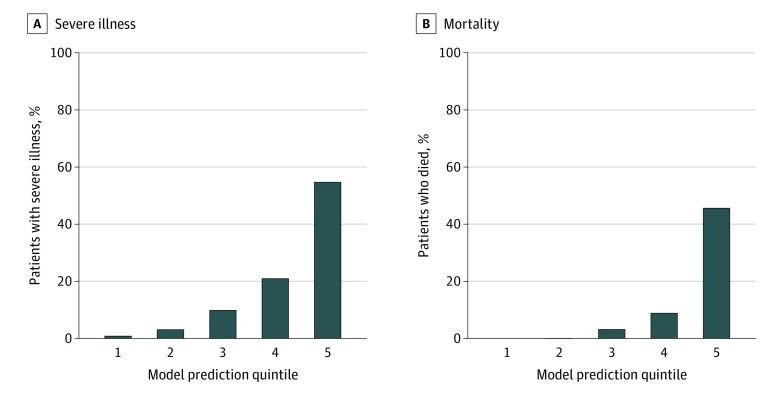
Quintile Plots of Outcomes in Independent Testing Cohort

In the independent testing set composed of a second academic medical center and 2 other community hospitals, the COVID-19–associated severe illness model yielded an area under the curve of 0.807 (Figure 1A), with a sensitivity of 60.6% and a specificity of 88.9% at the top risk quintile (the positive predictive value was 54.7%, whereas the negative predictive value was 91.1%). For the mortality model, the area under the curve was 0.847 (Figure 1B), with a sensitivity of 78.0% and a specificity of 87.5% (the positive predictive value was 45.6%, whereas the negative predictive value was 96.7%). Both models exhibited substantial lift, with the highest-risk quintile enriched for adverse outcomes in the test cohort (severe illness highest quintile lift: 3.0; mortality model highest quintile lift: 3.9) (Figure 2A and B).

**Figure 3.**
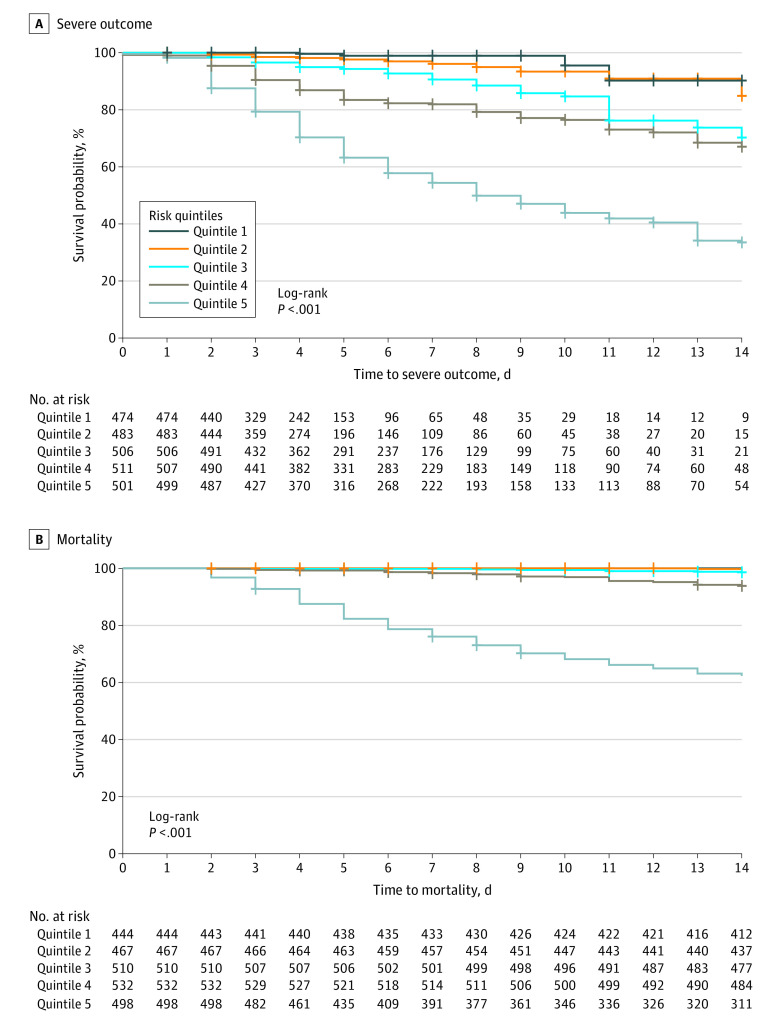
Kaplan-Meier Curves in Independent Testing Cohort

For illustrative purposes, we also examined the COVID-19–associated severe illness risk quintile (from the model incorporating all adverse outcomes) and mortality risk quintile in Kaplan-Meier survival curves, with curves censored at the time of hospital discharge, June 5, 2020 (ie, end of available follow-up), or 14 days, whichever came first (Figure 3). Quintiles were significantly associated with predicted outcome by log-rank test (χ^2^ = 818; *P* < .001). In total, 212 of 292 deaths (78%) occurred in the highest-risk mortality quintile.

## Discussion

In this study of 2511 individuals with COVID-19 hospitalized at academic medical centers and community hospitals in Eastern Massachusetts through June 5, 2020, 8.6% were admitted to the ICU, 6.5% required mechanical ventilation, and 11.6% died. In general, abnormal hematologic measures (including neutrophilia, lymphocytopenia, and eosinopenia), as well as diminished renal function, were associated with a greater risk of severe hospital course. Measures of prior pulmonary disease and of red blood cell abnormalities were also represented in the risk for mortality.

Discrimination of both models appears promising, identifying a high-risk quintile with reasonable sensitivity and specificity. Likewise, survival curves support the informativeness of the high-risk quintile and indicate that the results are not an artifact of differential attrition or shorter hospital stay. Predictions may be most useful during the initial week of hospitalization; a useful next-step study could examine whether rerunning models with additional laboratory studies, or incorporating other biomarkers, can improve longer-term prediction.

Our results are consistent with a recently reported study associating renal involvement with mortality.^17^ Multiple smaller cohorts have also reported laboratory features associated with morbidity and mortality among hospitalized patients with COVID-19. For example, a retrospective cohort study from Wuhan, China, of 191 hospitalized patients found that older age and a higher d-dimer level at admission were associated with risk of death.^6^

Among 95 fatal cases of COVID-19, low eosinophil count at admission was also common.^7^ An elevated ferritin level was also associated with mortality in a retrospective cohort study of 120 patients from Wuhan,^5^ along with elevated troponin and C-reactive peptide levels. Our results also confirm and extend those of the largest meta-analysis, to date, encompassing 3377 patients and implicating hematologic measures as well as renal function,^8^ in addition to markers of tissue injury more generally.

In developing these simple prediction models, we were mindful of the recent frameworks for and criticisms of such models,^10,18^ particularly the recognition that poorly validated or calibrated models may cause more harm than good. Initial models are likely optimistic (ie, overfit to data) and biased (ie, by nonrepresentative samples), with a lack of transparency.^18^ On the other hand, strategies that allow for risk stratification are particularly necessary in an environment of constrained resources. As such, we report these results in the hope that they will provide simple base-case models for others to improve on. Undoubtedly, the application of more complex models will yield further improvement in model fit, but whether the degree of improvement is sufficient to offset the added complexity of clinical implementation and reduced interpretability will merit careful consideration.

### Limitations

We note the multiple limitations that likely diminished model performance. First, because these are open hospital systems rather than closed networks, the lack of documented prior diagnoses does not preclude their presence for individuals who may receive care elsewhere. For this reason, we excluded hospital transfers because prior documentation of comorbidity is likely to be biased. However, such missing data are likely to diminish the predictive power of any given diagnosis, such that our model performance estimates are likely to be conservative. In addition, many laboratory values are highly nonnormal, such that incorporation of more specific transformations or cutpoints would likely improve model performance; we elected to incorporate standard high or low flags plus continuous measures, rather than adopting specific transformations for each value, which would risk overfitting or would diminish generalizability but likely extract additional information. Efforts to aggregate laboratory data across international health systems will provide an opportunity to explore such transformations if individual-level data become accessible.^9^

## Conclusions

The results of this cohort study suggest the utility of laboratory values in combination with documented comorbidities and sociodemographic features in identifying individuals at particularly high risk for more severe hospital course. Notably, by validating in distinct hospitals (albeit within a single geographic region), our estimates of model performance are likely to be less optimistic but still suggest that generalizability should be good. These admission models also provide an opportunity for comparison as more sophisticated models are developed, particularly those incorporating additional biological measures. To the extent hospital resources are constrained, the ability to target resources to highest-risk individuals is likely to be valuable, and expansion and refinement of risk models may represent a useful approach to optimizing care.

## Notes

Supplement. **eTable 1.** Lab Information **eTable 2.** Lab Value and Flag Comparisons in Training and Test Sets **eTable 3.** Severe Illness Model Unshrunk Coefficients **eTable 4.** Mortality Model Unshrunk Coefficients **eFigure.** Lab Distributions by Mortality Outcome
