# Supplementary material for: Laboratory Findings Associated With Severe Illness and Mortality Among Hospitalized Individuals With Coronavirus Disease 2019 in Eastern Massachusetts
Source: JAMA Netw Open. 2020 Oct 30;3(10):e2023934. doi: 10.1001/jamanetworkopen.2020.23934 (PMC7599467; doi:10.1001/jamanetworkopen.2020.23934)
Supplement: Supplement. — eTable 1. Lab Information eTable 2. Lab Value and Flag Comparisons in Training and Test Sets eTable 3. Severe Illness Model Unshrunk Coefficients eTable 4. Mortality Model Unshrunk Coefficients eFigure. Lab Distributions by Mortality Outcome [file jamanetwopen-e2023934-s001.pdf]

Supplemental Online Content

Castro VM, McCoy TH, Perlis RH. Laboratory findings associated with severe illness and mortality among hospitalized individuals with coronavirus disease 2019 in Eastern Massachusetts. *JAMA Netw Open*. 2020;3(10):e2023934. doi:10.1001/jamanetworkopen.2020.23934

eTable 1. Lab Information

eTable 2. Lab Value and Flag Comparisons in Training and Test Sets

eTable 3. Severe Illness Model Unshrunk Coefficients

eTable 4. Mortality Model Unshrunk Coefficients

eFigure. Lab Distributions by Mortality Outcome

This supplemental material has been provided by the authors to give readers additional information about their work.

© 2020 Castro VM et al. *JAMA Network Open.*

eTable 1: Lab Information

Lab panel	Lab code	LOINC	Lab name	Units
CBC and differential	BASOP	30180-4	BASOS (%)	%
CBC and differential	ABASOP	26444-0	BASOS, Absolute (K/uL)	K/uL
CBC and differential	EOSP	26450-7	EOS (%)	%
CBC and differential	AEOSN	26449-9	EOS, Absolute (K/uL)	K/uL
CBC and differential	IMMGRANP	71695-1	Granulocytes, Immature (%)	%
CBC and differential	IMMGRAN	51584-1	Granulocytes, Immature (K/uL)	K/uL
CBC and differential	HCT	4544-3	HCT (%)	%
CBC and differential	HGB	718-7	HGB (g/dL)	g/dL
CBC and differential	LYMP	26478-8	Lymphocytes (%)	%
CBC and differential	ALYMP	26474-7	Lymphocytes, Absolute (K/uL)	K/uL
CBC and differential	MCH	785-6	MCH (pg)	pg
CBC and differential	MCHC	786-4	MCHC (g/dL)	g/dL
CBC and differential	MCV	787-2	MCV (fL)	fL
CBC and differential	MON	26485-3	Monocytes (%)	%
CBC and differential	AMONS	26484-6	Monocytes, Absolute (K/uL)	K/uL
CBC and differential	MVP	776-5	MPV (fL)	fl
CBC and differential	NEUT	26511-6	Neutrophils (%)	%
CBC and differential	ANEU	26499-4	Neutrophils, Absolute (K/uL)	K/uL
CBC and differential	NRBC	19048-8	NRBC% (/100 WBC)	/100 WBCs
CBC and differential	NRBCA	771-6	NRBC, Absolute (K/uL)	K/uL
CBC and differential	PLT	777-3	PLT (K/uL)	K/uL
CBC and differential	RBC	789-8	RBC (M/uL)	M/uL
CBC and differential	RDW	788-0	RDW (%)	%
CBC and differential	WBC	6690-2	WBC (K/uL)	K/uL
Hepatic panel	ALB	1751-7	Albumin (g/dL)	g/dL
Hepatic panel	SGPT	1742-6	ALT (U/L)	U/L
Hepatic panel	SGOT	1920-8	AST (U/L)	U/L
Hepatic panel	DBILI	1968-7	Bilirubin, Direct (mg/dL)	mg/dL
Hepatic panel	TBILI	1975-2	Bilirubin, Total (mg/dL)	mg/dL
Hepatic panel	GLOB	2336-6	Globulin (g/dL)	g/dL
Hepatic panel	TP	2885-2	Protein, Total (g/dL)	g/dL
Metabolic panel	ALKP	6768-6	Alkaline Phosphatase (U/L)	U/L
Metabolic panel	ANION	10466-1	Anion Gap (mmol/L)	mmol/L
Metabolic panel	BUN	3094-0	BUN (mg/dL)	mg/dL
Metabolic panel	CA	17861-6	Calcium (mg/dL)	mg/dL
Metabolic panel	CO2	2028-9	Carbon dioxide (mmol/L)	mmol/L
Metabolic panel	CL	2075-0	Chloride (mmol/L)	mmol/L
Metabolic panel	CPK	2157-6	Creatinine Kinase (U/L)	U/L
Metabolic panel	GFR	33914-3	GFR, estimated (mL/min/1.73m2)	mL/min/1.73m2
Metabolic panel	GLU	2345-7	Glucose (mg/dL)	mg/dL
Metabolic panel	K	2823-3	Potassium (mmol/L)	mmol/L
Metabolic panel	NA	2951-2	Sodium (mmol/L)	mmol/L
Additional Labs	CRPT	1988-5	C-Reactive Protein (mg/L)	mg/L
Additional Labs	DDIMER*	48058-2	D-DIMER (ng/ml)	ng/mL
Additional Labs	ESR	4537-7	ESR (mm/h)	mm/h
Additional Labs	FER	2276-4	Ferritin (ug/L)	ug/L
Additional Labs	LACT	2524-7	Lactic Acid (mmol/L)	mmol/L
Additional Labs	LDH	2532-0	LDH (U/L)	U/L
Additional Labs	MG	2601-3	Magnesium (mg/dL)	mg/dL
Additional Labs	PRCCITON	33959-8	Procalcitonin (ng/mL)	ng/mL
Additional Labs	TROPTHS	67151-1	Troponin T (ng/L)	ng/L

** Some results were reported in different units and converted to normal units shown*

© 2020 Castro VM et al. *JAMA Network Open.*

eTable 2: Lab value and flag comparisons in training and test sets

	Train (N=1877)	Test (N=634)	Total (N=2511)	p value
**C-Reactive Protein (mg/L)**				0.036
N-Miss	139	117	256	
Median (Q1, Q3)	63.900 (25.700, 124.700)	74.100 (18.600, 133.300)	65.300 (25.100, 126.750)	
High Flag	1568 (83.5%)	454 (71.6%)	2022 (80.5%)	
**DDIMER**				0.069
N-Miss	206	88	294	
Median (Q1, Q3)	904.000 (580.000, 1570.000)	1002.500 (577.250, 1960.250)	918.000 (580.000, 1668.000)	
High	1367 (72.8%)	434 (68.5%)	1801 (71.7%)	
**ESR (mm/h)**				< 0.001
N-Miss	401	267	668	
Median (Q1, Q3)	42.000 (25.000, 67.000)	51.000 (31.000, 79.000)	44.000 (26.000, 69.000)	
High	1272 (67.8%)	319 (50.3%)	1591 (63.4%)	
High Panic	1 (0.1%)	0 (0.0%)	1 (0.0%)	
**Ferritin (ug/L)**				0.655
N-Miss	139	95	234	
Median (Q1, Q3)	506.000 (241.250, 933.500)	411.000 (200.500, 913.000)	495.000 (227.000, 929.000)	
High	1293 (68.9%)	375 (59.1%)	1668 (66.4%)	
Low	5 (0.3%)	5 (0.8%)	10 (0.4%)	
**LDH (U/L)**				0.040
N-Miss	141	93	234	
Median (Q1, Q3)	277.500 (219.000, 356.000)	283.000 (225.000, 379.000)	279.000 (221.000, 360.000)	
High	1316 (70.1%)	397 (62.6%)	1713 (68.2%)	
Low	3 (0.2%)	8 (1.3%)	11 (0.4%)	
**Magnesium (mg/dL)**				0.748
N-Miss	337	46	383	
Median (Q1, Q3)	2.000 (1.900, 2.200)	2.000 (1.900, 2.200)	2.000 (1.900, 2.200)	
High	55 (2.9%)	15 (2.4%)	70 (2.8%)	
Low	93 (5.0%)	37 (5.8%)	130 (5.2%)	
Low Panic	1 (0.1%)	0 (0.0%)	1 (0.0%)	
**Procalcitonin (ng/mL)**				0.021
N-Miss	289	69	358	
Median (Q1, Q3)	0.130 (0.080, 0.230)	0.120 (0.060, 0.260)	0.120 (0.080, 0.240)	
High	1129 (60.1%)	316 (49.8%)	1445 (57.5%)	
**Troponin T (ng/L)**				0.040
N-Miss	188	79	267	
Median (Q1, Q3)	12.000 (6.000, 31.000)	12.000 (7.000, 26.000)	12.000 (6.000, 29.000)	
High	862 (45.9%)	286 (45.1%)	1148 (45.7%)	
**BASOS (%)**				< 0.001
N-Miss	115	19	134	
Median (Q1, Q3)	0.300 (0.200, 0.500)	0.200 (0.100, 0.400)	0.300 (0.200, 0.400)	
High	12 (0.6%)	2 (0.3%)	14 (0.6%)	

© 2020 Castro VM et al. *JAMA Network Open.*

**BASOS, Absolute (K/uL)**				< 0.001
N-Miss	115	19	134	
Median (Q1, Q3)	0.020 (0.010, 0.030)	0.010 (0.010, 0.020)	0.020 (0.010, 0.030)	
High	2 (0.1%)	4 (0.6%)	6 (0.2%)	
**EOS (%)**				0.002
N-Miss	89	17	106	
Median (Q1, Q3)	0.500 (0.000, 1.600)	0.300 (0.000, 1.200)	0.400 (0.000, 1.500)	
High	58 (3.1%)	16 (2.5%)	74 (2.9%)	
**EOS, Absolute (K/uL)**				0.002
N-Miss	89	17	106	
Median (Q1, Q3)	0.030 (0.000, 0.090)	0.020 (0.000, 0.070)	0.030 (0.000, 0.090)	
High	24 (1.3%)	4 (0.6%)	28 (1.1%)	
Low	304 (16.2%)	0 (0.0%)	304 (12.1%)	
**Granulocytes, Immature (%)**				0.466
N-Miss	172	63	235	
Median (Q1, Q3)	0.500 (0.300, 0.800)	0.500 (0.300, 0.800)	0.500 (0.300, 0.800)	
High	219 (11.7%)	97 (15.3%)	316 (12.6%)	
**Granulocytes, Immature (K/uL)**				0.580
N-Miss	172	63	235	
Median (Q1, Q3)	0.030 (0.020, 0.050)	0.030 (0.020, 0.060)	0.030 (0.020, 0.050)	
High	178 (9.5%)	74 (11.7%)	252 (10.0%)	
**HCT (%)**				0.003
Median (Q1, Q3)	37.400 (33.500, 40.700)	36.500 (33.025, 39.800)	37.100 (33.400, 40.500)	
High	17 (0.9%)	4 (0.6%)	21 (0.8%)	
High Panic	1 (0.1%)	0 (0.0%)	1 (0.0%)	
Low	1021 (54.4%)	353 (55.7%)	1374 (54.7%)	
Low Panic	1 (0.1%)	1 (0.2%)	2 (0.1%)	
**HGB (g/dL)**				< 0.001
Median (Q1, Q3)	12.300 (10.800, 13.500)	11.800 (10.600, 13.000)	12.100 (10.750, 13.400)	
High	5 (0.3%)	3 (0.5%)	8 (0.3%)	
Low	1101 (58.7%)	368 (58.0%)	1469 (58.5%)	
Low Panic	0 (0.0%)	1 (0.2%)	1 (0.0%)	
**Lymphocytes (%)**				0.108
N-Miss	35	16	51	
Median (Q1, Q3)	19.550 (12.800, 27.700)	20.850 (13.400, 29.150)	19.850 (13.000, 28.100)	
High	118 (6.3%)	47 (7.4%)	165 (6.6%)	
Low	886 (47.2%)	255 (40.2%)	1141 (45.4%)	
**Lymphocytes, Absolute (K/uL)**				0.149
N-Miss	35	16	51	
Median (Q1, Q3)	1.140 (0.790, 1.590)	1.190 (0.770, 1.660)	1.150 (0.780, 1.610)	
High	11 (0.6%)	8 (1.3%)	19 (0.8%)	
Low	747 (39.8%)	151 (23.8%)	898 (35.8%)	
**MCH (pg)**				0.248
Median (Q1, Q3)	29.200 (27.900, 30.400)	29.100 (27.400, 30.700)	29.200 (27.800, 30.400)	
High	100 (5.3%)	52 (8.2%)	152 (6.1%)	
Low	222 (11.8%)	126 (19.9%)	348 (13.9%)	

© 2020 Castro VM et al. *JAMA Network Open.*

**MCHC (g/dL)**				< 0.001
Median (Q1, Q3)	32.900 (31.800, 33.700)	32.450 (31.425, 33.300)	32.800 (31.700, 33.600)	
High	7 (0.4%)	8 (1.3%)	15 (0.6%)	
Low	364 (19.4%)	224 (35.3%)	588 (23.4%)	
**MCV (fL)**				0.021
Median (Q1, Q3)	88.300 (84.800, 92.100)	88.900 (84.900, 93.700)	88.500 (84.800, 92.500)	
High	79 (4.2%)	61 (9.6%)	140 (5.6%)	
Low	136 (7.2%)	44 (6.9%)	180 (7.2%)	
**Monocytes (%)**				0.101
N-Miss	37	16	53	
Median (Q1, Q3)	7.700 (5.400, 10.200)	7.900 (5.600, 10.700)	7.700 (5.500, 10.300)	
High	251 (13.4%)	138 (21.8%)	389 (15.5%)	
Low	215 (11.5%)	68 (10.7%)	283 (11.3%)	
**Monocytes, Absolute (K/uL)**				0.066
N-Miss	37	16	53	
Median (Q1, Q3)	0.440 (0.290, 0.630)	0.480 (0.320, 0.650)	0.450 (0.300, 0.640)	
High	74 (3.9%)	33 (5.2%)	107 (4.3%)	
Low	156 (8.3%)	38 (6.0%)	194 (7.7%)	
**MPV (fL)**				< 0.001
N-Miss	41	14	55	
Median (Q1, Q3)	10.300 (9.700, 11.000)	10.600 (9.900, 11.200)	10.400 (9.800, 11.100)	
High	113 (6.0%)	66 (10.4%)	179 (7.1%)	
Low	57 (3.0%)	2 (0.3%)	59 (2.3%)	
**Neutrophils (%)**				0.711
N-Miss	35	16	51	
Median (Q1, Q3)	68.650 (58.700, 77.400)	67.600 (58.075, 77.700)	68.300 (58.500, 77.400)	
High	773 (41.2%)	187 (29.5%)	960 (38.2%)	
Low	125 (6.7%)	65 (10.3%)	190 (7.6%)	
**Neutrophils, Absolute (K/uL)**				0.997
N-Miss	35	16	51	
Median (Q1, Q3)	4.000 (2.740, 5.648)	4.030 (2.675, 5.603)	4.000 (2.718, 5.640)	
High	226 (12.0%)	79 (12.5%)	305 (12.1%)	
Low	138 (7.4%)	67 (10.6%)	205 (8.2%)	
Low Panic	0 (0.0%)	1 (0.2%)	1 (0.0%)	
**NRBC% (/100 WBC)**				NaN
Median (Q1, Q3)	0.000 (0.000, 0.000)	0.000 (0.000, 0.000)	0.000 (0.000, 0.000)	
High	53 (2.8%)	29 (4.6%)	82 (3.3%)	
**NRBC, Absolute (K/uL)**				NaN
N-Miss	361	4	365	
Median (Q1, Q3)	0.000 (0.000, 0.000)	0.000 (0.000, 0.000)	0.000 (0.000, 0.000)	
High	50 (2.7%)	28 (4.4%)	78 (3.1%)	
**PLT (K/uL)**				0.936
N-Miss	4	0	4	
Median (Q1, Q3)	203.000 (155.000, 265.000)	199.500 (154.250, 267.000)	203.000 (155.000, 265.000)	
High	89 (4.7%)	12 (1.9%)	101 (4.0%)	
Low	387 (20.6%)	135 (21.3%)	522 (20.8%)	
Low Panic	6 (0.3%)	4 (0.6%)	10 (0.4%)	

© 2020 Castro VM et al. *JAMA Network Open.*

**RBC (M/uL)**				< 0.001
Median (Q1, Q3)	4.250 (3.740, 4.680)	4.115 (3.680, 4.530)	4.210 (3.725, 4.650)	
High	40 (2.1%)	3 (0.5%)	43 (1.7%)	
Low	885 (47.1%)	308 (48.6%)	1193 (47.5%)	
**RDW (%)**				< 0.001
N-Miss	1	0	1	
Median (Q1, Q3)	13.500 (12.800, 14.600)	13.950 (13.000, 15.200)	13.600 (12.800, 14.700)	
High	484 (25.8%)	226 (35.6%)	710 (28.3%)	
Low	17 (0.9%)	5 (0.8%)	22 (0.9%)	
**WBC (K/uL)**				0.589
Median (Q1, Q3)	5.940 (4.520, 7.790)	5.895 (4.470, 7.950)	5.920 (4.510, 7.840)	
High	151 (8.0%)	70 (11.0%)	221 (8.8%)	
High Panic	0 (0.0%)	1 (0.2%)	1 (0.0%)	
Low	402 (21.4%)	98 (15.5%)	500 (19.9%)	
Low Panic	0 (0.0%)	6 (0.9%)	6 (0.2%)	
**Albumin (g/dL)**				0.947
N-Miss	84	20	104	
Median (Q1, Q3)	3.400 (3.100, 3.700)	3.400 (3.100, 3.700)	3.400 (3.100, 3.700)	
High	2 (0.1%)	0 (0.0%)	2 (0.1%)	
Low	781 (41.6%)	330 (52.1%)	1111 (44.2%)	
**ALT (U/L)**				0.022
N-Miss	84	25	109	
Median (Q1, Q3)	25.000 (15.000, 43.000)	23.000 (14.000, 38.000)	24.000 (15.000, 42.000)	
High	401 (21.4%)	100 (15.8%)	501 (20.0%)	
Low	130 (6.9%)	41 (6.5%)	171 (6.8%)	
**AST (U/L)**				0.038
N-Miss	84	25	109	
Median (Q1, Q3)	34.000 (24.000, 52.000)	32.000 (23.000, 49.000)	34.000 (24.000, 51.000)	
High	748 (39.9%)	150 (23.7%)	898 (35.8%)	
Low	35 (1.9%)	3 (0.5%)	38 (1.5%)	
**Bilirubin, Direct (mg/dL)**				< 0.001
N-Miss	207	50	257	
Median (Q1, Q3)	0.200 (0.200, 0.200)	0.200 (0.200, 0.200)	0.200 (0.200, 0.200)	
High	81 (4.3%)	36 (5.7%)	117 (4.7%)	
Low	0 (0.0%)	1 (0.2%)	1 (0.0%)	
**Bilirubin, Total (mg/dL)**				0.424
N-Miss	84	20	104	
Median (Q1, Q3)	0.400 (0.300, 0.600)	0.400 (0.300, 0.500)	0.400 (0.300, 0.600)	
High	73 (3.9%)	28 (4.4%)	101 (4.0%)	
**Globulin (g/dL)**				0.001
N-Miss	85	20	105	
Median (Q1, Q3)	3.400 (3.100, 3.800)	3.300 (2.900, 3.800)	3.400 (3.000, 3.800)	
High	197 (10.5%)	57 (9.0%)	254 (10.1%)	
Low	3 (0.2%)	10 (1.6%)	13 (0.5%)	
**Protein, Total (g/dL)**				0.010
N-Miss	85	20	105	
Median (Q1, Q3)	6.900 (6.400, 7.300)	6.800 (6.300, 7.300)	6.900 (6.400, 7.300)	
High	40 (2.1%)	21 (3.3%)	61 (2.4%)	

© 2020 Castro VM et al. *JAMA Network Open.*

Low	160 (8.5%)	150 (23.7%)	310 (12.3%)	
**Alkaline Phosphatase (U/L)**				0.547
N-Miss	85	22	107	
Median (Q1, Q3)	74.000 (58.000, 96.000)	71.000 (57.000, 96.000)	74.000 (57.000, 96.000)	
High	299 (15.9%)	68 (10.7%)	367 (14.6%)	
Low	38 (2.0%)	19 (3.0%)	57 (2.3%)	
**Anion Gap (mmol/L)**				< 0.001
N-Miss	37	12	49	
Median (Q1, Q3)	13.000 (12.000, 15.000)	14.000 (12.000, 16.000)	14.000 (12.000, 15.000)	
High	107 (5.7%)	64 (10.1%)	171 (6.8%)	
Low	0 (0.0%)	1 (0.2%)	1 (0.0%)	
**BUN (mg/dL)**				0.388
N-Miss	35	12	47	
Median (Q1, Q3)	14.000 (10.000, 22.000)	15.000 (10.000, 23.000)	14.000 (10.000, 22.000)	
High	428 (22.8%)	157 (24.8%)	585 (23.3%)	
Low	194 (10.3%)	25 (3.9%)	219 (8.7%)	
**Calcium (mg/dL)**				< 0.001
N-Miss	35	12	47	
Median (Q1, Q3)	8.800 (8.500, 9.100)	8.600 (8.300, 9.000)	8.800 (8.400, 9.100)	
High	10 (0.5%)	2 (0.3%)	12 (0.5%)	
Low	646 (34.4%)	314 (49.5%)	960 (38.2%)	
Low Panic	1 (0.1%)	0 (0.0%)	1 (0.0%)	
**Carbon dioxide (mmol/L)**				0.475
N-Miss	36	12	48	
Median (Q1, Q3)	24.000 (22.000, 26.000)	24.000 (22.000, 26.000)	24.000 (22.000, 26.000)	
High	17 (0.9%)	12 (1.9%)	29 (1.2%)	
Low	476 (25.4%)	148 (23.3%)	624 (24.9%)	
**Chloride (mmol/L)**				0.678
N-Miss	35	12	47	
Median (Q1, Q3)	101.000 (98.000, 104.000)	101.000 (98.000, 104.000)	101.000 (98.000, 104.000)	
High	177 (9.4%)	59 (9.3%)	236 (9.4%)	
Low	303 (16.1%)	125 (19.7%)	428 (17.0%)	
**Creatinine Kinase (U/L)**				0.969
N-Miss	171	89	260	
Median (Q1, Q3)	99.000 (55.000, 208.750)	95.000 (51.000, 198.000)	99.000 (54.000, 205.500)	
High	324 (17.3%)	122 (19.2%)	446 (17.8%)	
High Panic	0 (0.0%)	1 (0.2%)	1 (0.0%)	
Low	250 (13.3%)	80 (12.6%)	330 (13.1%)	
**GFR, estimated (mL/min/1.73m2)**				0.017
N-Miss	33	13	46	
Median (Q1, Q3)	85.000 (57.000, 103.000)	80.000 (54.000, 98.000)	84.000 (57.000, 102.000)	
Low	496 (26.4%)	181 (28.5%)	677 (27.0%)	
**Glucose (mg/dL)**				0.753
N-Miss	33	12	45	
Median (Q1, Q3)	109.000 (97.000, 133.000)	107.000 (93.000, 134.750)	109.000 (96.000, 133.000)	
High	1026 (54.7%)	334 (52.7%)	1360 (54.2%)	
High Panic	1 (0.1%)	1 (0.2%)	2 (0.1%)	

© 2020 Castro VM et al. *JAMA Network Open.*

Low	22 (1.2%)	6 (0.9%)	28 (1.1%)	
Low Panic	1 (0.1%)	0 (0.0%)	1 (0.0%)	
**Potassium (mmol/L)**				0.595
N-Miss	37	14	51	
Median (Q1, Q3)	4.000 (3.700, 4.300)	4.000 (3.700, 4.300)	4.000 (3.700, 4.300)	
High	25 (1.3%)	8 (1.3%)	33 (1.3%)	
High Panic	3 (0.2%)	0 (0.0%)	3 (0.1%)	
Low	152 (8.1%)	35 (5.5%)	187 (7.4%)	
Low Panic	4 (0.2%)	0 (0.0%)	4 (0.2%)	

© 2020 Castro VM et al. *JAMA Network Open.*

eTable 3: Severe illness model unshrunk coefficients

Feature	Coefficient	95% CI Low	95% CI High
(Intercept)	-1.5015	-5.3984	2.2352
Age at admission	0.0249	0.0146	0.0354
Baseline O2Sat	-0.0364	-0.0648	-0.0067
Charlson comorbidity index	0.0514	0.0011	0.1012
Lymphocytes (%) (continuous)	-0.0137	-0.0473	0.0202
Neutrophils (%) (continuous)	-0.0104	-0.0359	0.0156
EOS (%) (continuous)	-0.6978	-0.9156	-0.4985
C-reactive protein (mg/L) (continuous)	0.0039	0.0015	0.0064
LDH (U/L) (continuous)	0.0052	0.0038	0.0067
Platelets (K/uL) (continuous)	-0.0040	-0.0067	-0.0014
Troponin T (ng/dL) (continuous)	0.0079	0.0016	0.0142
Glucose (mg/dL) (continuous)	0.0067	0.0031	0.0103
BUN (mg/dL) (continuous)	0.0005	-0.0177	0.0187
Prior diagnosis of respiratory infections (CCS 8.1)	0.2598	0.1255	0.3940
Neutrophils, absolute (High)	0.5716	0.1414	1.0019
Monocytes (Low)	0.5515	0.1450	0.9538
Lymphocytes, absolute (Low)	0.4969	0.1408	0.8548
Platelets (Low)	0.1801	-0.2386	0.5974
eGFR (Low)	0.0992	-0.3988	0.5872
Creatinine (High)	0.4348	-0.1105	0.9859

© 2020 Castro VM et al. *JAMA Network Open.*

eTable 4: Mortality model unshrunk coefficients

Feature	Coefficient	95% CI Low	95% CI High
(Intercept)	-6.7340		
NRBC, absolute (High)	0.9345	0.0118	1.8649
Platelets (Low)	0.7197	0.3114	1.1274
WBC (High)	0.6404	-0.1337	1.4107
Creatinine (High)	0.6048	-0.0336	1.2538
Lymphocytes, absolute (Low)	0.5467	0.0610	1.0395
Procalcitonin (ng/mL) (continuous)	0.4680	-0.0261	0.9548
MCHC (Low)	0.4201	-0.0169	0.8526
Neutrophils, absolute (High)	0.3173	-0.3526	0.9748
Prior diagnosis of lung cancer (CCS 2.3)	0.3019	-0.0038	0.6261
Prior diagnosis of external causes of injury (CCS 18)	0.1907	0.0283	0.3542
Prior diagnosis of respiratory failure/insufficiency (CCS 8.6)	0.1659	-0.0931	0.4191
RDW (%) (continuous)	0.1174	-0.0185	0.2515
Prior diagnosis of COPD/bronchiectasis (CCS 8.2)	0.0953	-0.0939	0.2821
Age at admission	0.0664	0.0500	0.0838
Prior diagnosis of dementia/delirium (CCS 5.4)	0.0615	-0.1077	0.2277
Charlson comorbidity index	0.0127	-0.0598	0.0836
BUN (mg/dL) (continuous)	0.0102	-0.0111	0.0313
LDH (U/L) (continuous)	0.0042	0.0023	0.0061
Troponin T (ng/dL) (continuous)	0.0024	-0.0053	0.0101
Lymphocytes (%) (continuous)	-0.0025	-0.0306	0.0249
eGFR (Low)	-0.0263	-0.6209	0.5527
Baseline O2Sat	-0.0479	-0.0797	-0.0124
EOS (%) (continuous)	-0.8193	-1.1151	-0.5564

© 2020 Castro VM et al. *JAMA Network Open.*

eFigure: Lab distributions by mortality outcome

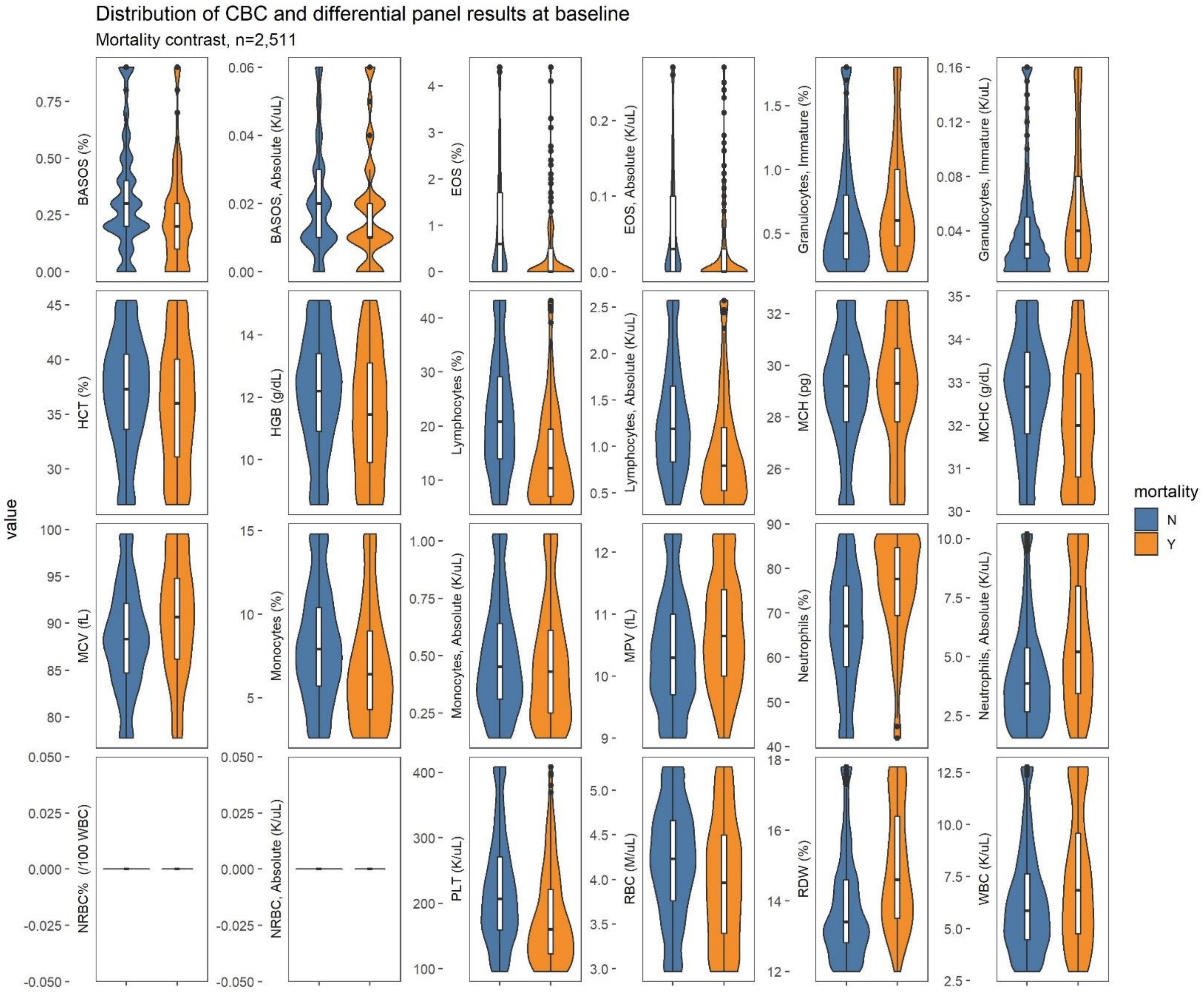

© 2020 Castro VM et al. *JAMA Network Open.*

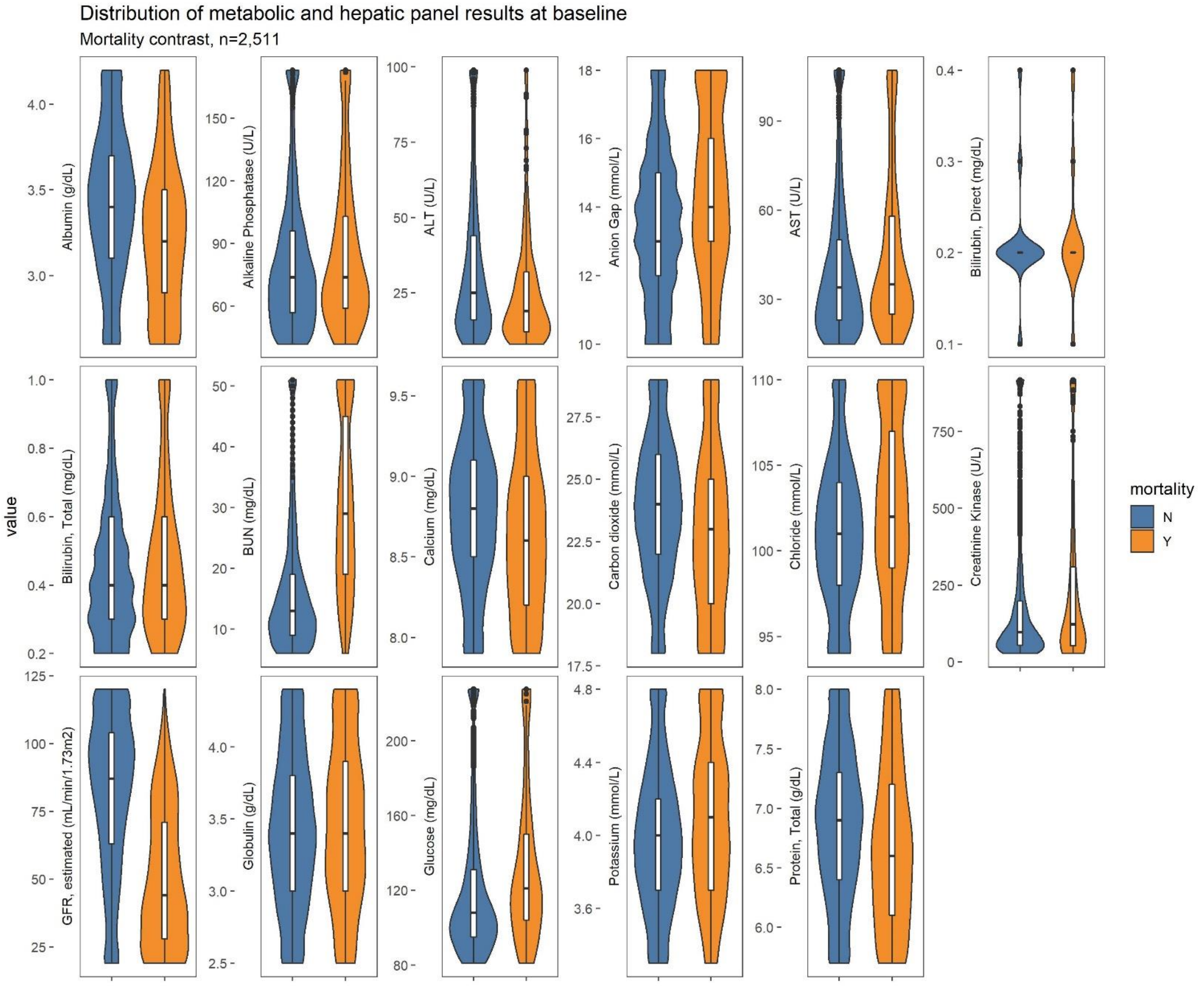

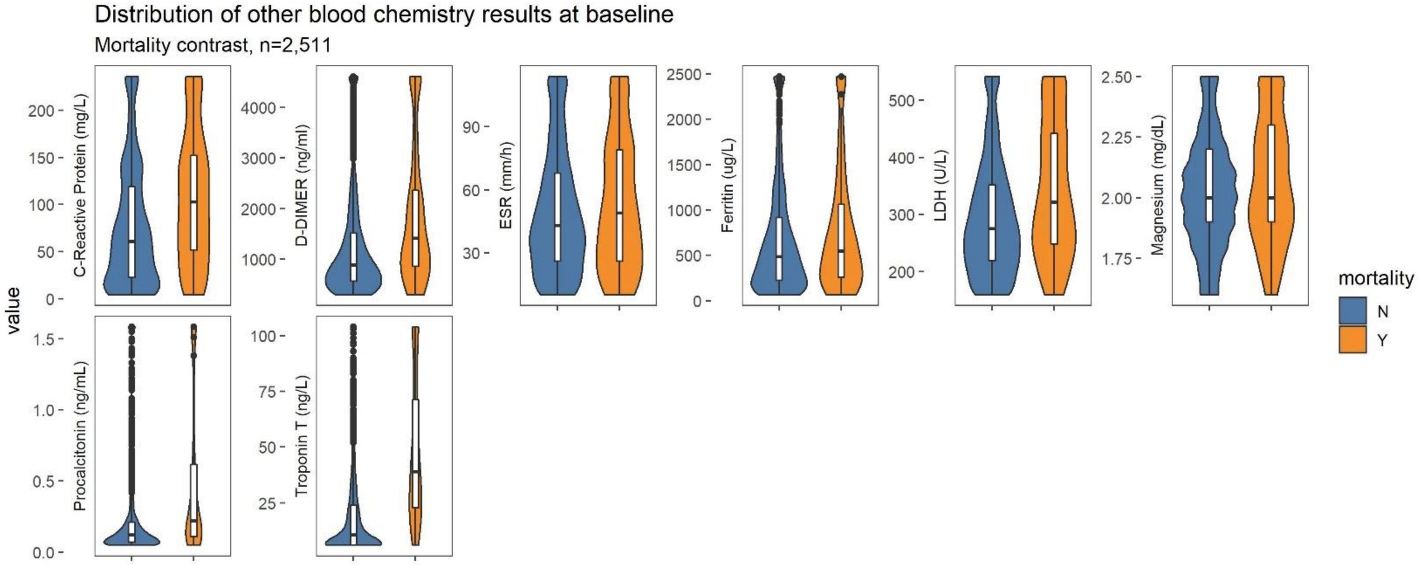

© 2020 Castro VM et al. *JAMA Network Open.*
